# Automated synthesis, preclinical toxicity, and radiation dosimetry of [^18^F]MC225 for clinical use: a tracer for measuring P-glycoprotein function at the blood-brain barrier

**DOI:** 10.1186/s13550-020-00674-6

**Published:** 2020-07-22

**Authors:** Jun Toyohara, Muneyuki Sakata, Tetsuro Tago, Nicola A. Colabufo, Gert Luurtsema

**Affiliations:** 1 Research Team for Neuroimaging, Tokyo Metropolitan Institute of Gerontology, Tokyo, Japan; 2 Dipartimento di Farmacia-Scienze del Farmaco, Università degli Studi di Bari Aldo Moro, Bari, Italy; 3 Department of Nuclear Medicine and Molecular Imaging, University of Groningen and University Medical Centre Groningen, Groningen, The Netherlands

**Keywords:** Positron emission tomography, P-glycoprotein, MC225, Fluorine-18, Dosimetry, Toxicology

## Abstract

**Introduction:**

[^18^F]MC225 is a selective substrate for P-glycoprotein (P-gp) that has good metabolic stability and shows higher baseline uptake compared with other P-gp substrates such as (*R*)-[^11^C]Verapamil. Prior to clinical translation, it is necessary to perform process validation of the radiosynthesis, assessment of preclinical toxicity, and radiation dosimetry.

**Methods:**

The production of [^18^F]MC225 was automated on a CFN-MPS200 multipurpose synthesizer. The acute toxicity of MC225 was evaluated at a dose of 2.5 mg/kg bodyweight, which is more than 10,000-fold the postulated maximum clinical dose of [^18^F]MC225. The acute toxicity of [^18^F]MC225 injection at a 200-fold dose, to administer a postulated dose of 185 MBq of [^18^F]MC225, was also evaluated after the decay-out of ^18^F. The mutagenicity of MC225 was studied by a reverse mutation test using *Salmonella typhimurium* and *Escherichia coli* (Ames test). In vivo biodistribution and dosimetry studies of [^18^F]MC225 were carried out in normal mice. Human dosimetry was estimated using OLINDA software.

**Results:**

The mean decay-corrected yields of [^18^F]MC225 at end of synthesis were 13%, with > 99% radiochemical purity, > 1000 GBq/μmol molar activity, and ≤ 1.5 μg/185 MBq of total chemical contents. All process validation batches complied with the product specifications and the process was confirmed to be appropriate for the production of [^18^F]MC225. No acute toxicity of MC225 or [^18^F]MC225 injection was found. No mutagenic activity was observed for MC225. The biodistribution study demonstrated both hepatobiliary and renal excretion of radioactivity. The most critical organ was the pancreas, with (63.8 μGy/MBq) or without urination (63.9 μGy/MBq) at 360 min after injection. The estimated effective dose (μSv/MBq) with and without urination at 360 min after injection was calculated as 15.7 and 16.9, respectively.

**Conclusion:**

[^18^F]MC225 shows acceptable pharmacological safety at the dose required for adequate PET imaging. The potential risk associated with [^18^F]MC225 PET imaging is well within acceptable dose limits.

## Introduction

The blood-brain barrier (BBB) plays an important role in protecting the brain from xenobiotics and in maintaining homeostasis in the internal environment of the central nervous system (CNS) [1]. P-glycoprotein (P-gp) is an ATP-binding cassette transporter that is constitutively expressed in the luminal membrane of the BBB. It protects brain tissue against small hydrophobic xenobiotics that can passively diffuse through the BBB by selectively transporting them from cells into the extracellular space [2]. Hence, P-gp may also limit or prevent access of drugs such as antiepileptics, antidepressants, and anticancer agents to their target site in the brain [3]. Multiple clinical and preclinical evidence suggests that enhanced P-gp function at the BBB may be responsible for drug resistance in several diseases, including epilepsy [4–8], depression [9, 10], and human immunodeficiency virus infection and acquired immune deficiency syndrome [11–13]. Furthermore, altered P-gp function at the BBB has been proposed as a possible etiology of neurodegenerative disease; for example, decreased P-gp function may decrease clearance of β-amyloid from interstitial fluid in the brain to the plasma, which would result in a predisposition for β-amyloid deposition in Alzheimer’s disease [14–20]. A significant decrease of P-gp function in Parkinson’s disease patients is likely to facilitate the accumulation of toxic compounds in the brain [21–23].

Several potent P-gp substrates, including (*R*)-Verapamil, have been labeled for imaging P-gp function with positron emission tomography (PET) [24, 25]. These substrates have high affinity for P-gp and measure decreased function as increased tracer uptake by the brain. However, they are not likely to measure overexpression of P-gp because the concentration of the tracer is already almost unmeasurable at baseline [24–27]. Besides low brain uptake of (*R*)-[^11^C]verapamil, another disadvantage of this radiotracer is the formation of labeled metabolites, which also act as P-gp substrates [28].

5-(1-(2-[^18^F]fluoroethoxy))-[3-(6,7-dimethoxy-3,4-dihydro-1H-isoquinolin-2-yl)-propyl]-5,6,7,8-tetrahydronaphthalen ([^18^F]MC225) has recently been developed as a selective substrate for P-gp with good metabolic stability and has shown higher baseline uptake than that of other P-gp substrates [29, 30] because it is a weak substrate. These are suitable properties for measuring overexpression of P-gp in the brain. Preclinical studies have proved that [^18^F]MC225 has sufficient sensitivity to detect daily fluctuation of P-gp function in the rodent brain [31]. Very recently, a head-to-head comparison of [^18^F]MC225 with (*R*)-[^11^C]Verapamil in non-human primates showed a higher baseline uptake of [^18^F]MC225, which makes it a suitable tracer for measuring overexpression of P-gp [32]. These findings prompted us to undertake initial evaluation of [^18^F]MC225 in human subjects as a phase 1 study. As the first step prior to clinical application in humans, we performed a process validation of [^18^F]MC225 radiosynthesis for clinical use and assessed the preclinical toxicity and radiation dosimetry estimated from mouse distribution data.

Parts of this study have been published as a poster in 2019 [33].

## Materials and methods

### General

MC225 and its phenol precursor, 5-(1-(2-hydroxy))-[3-(6,7-dimethoxy-3,4-dihydro-1H-isoquinolin-2-yl)-propyl]-5,6,7,8-tetrahydronaphthalen (MC226) was custom synthesized by Nard Institute (Kobe, Japan) according to the methods described previously [29]. 2-Bromoethyl tosylate was purchased from ABX (Radeberg, Germany). All other chemical reagents were obtained from commercial sources. Male ddY mice were obtained from Japan SLC (Shizuoka, Japan). Sprague-Dawley [Crl:CD(SD)] rats were obtained from Charles River Laboratories (Atsugi, Japan). The animal studies were approved by the Animal Care and Use committee of the Tokyo Metropolitan Institute of Gerontology (Approval Nos. 17079, 17080, and 18016) and BoZo Research Center (Approval Nos. K180026 and K180027). Acute toxicity studies were performed under the guidelines of the “Revision of Guidelines for Single-Dose and Repeated-Dose Toxicity Studies”, Notification No. 88 of the Pharmaceutical Affairs Bureau, Ministry of Health and Welfare, Japan, August 10, 1993. Ames test was performed under the “Guidelines for Genotoxicity Studies of Pharmaceuticals” Yakushoku-shinsa No. 0920-1: September 20, 2012, Pharmaceutical and Food Safety Bureau, Ministry of Health, Labour and Welfare (MHLW).

### Automated synthesis

#### Setup of automated synthesizer

**Fig. 1:**
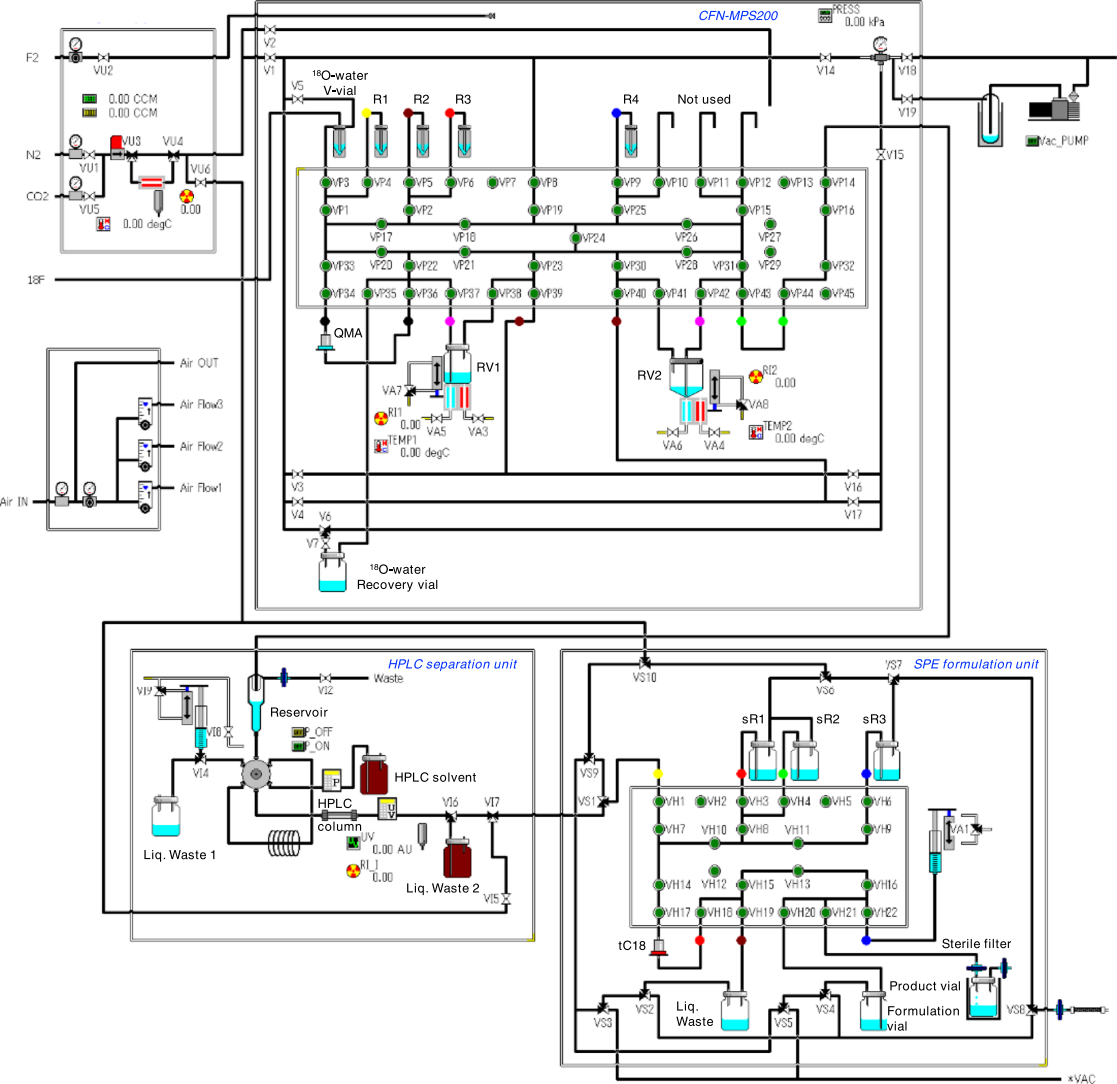
Setup of the synthesis cassette, reagents, and vials for [^18^F]MC225 production with integrated HPLC separation and SPE formulation units

[^18^F]MC225 was produced on a CFN-MPS200 multipurpose synthesizer (Sumitomo Heavy Industries, Tokyo, Japan) with a custom-made disposable cassette and an integrated high-performance liquid chromatography (HPLC) purification unit and solid-phase extraction (SPE) formulation unit with a sterile disposable cassette. Figure 1 shows the cassette setup, including tubing connections, vials, and other disposables. The custom-made cassettes were pre-assembled in a cleanroom using disposable materials supplied by Sumitomo Heavy Industries. We selected PharMed® BOT tube (Saint-Gobain, Akron, OH, USA) because of its good general chemical resistance and excellent acid, alkali, and oxidation resistance. Table 1 lists the module setup of reagents in detail. A Sep-Pak Accell Plus QMA Light Cartridge (Waters, Milford, MA, USA) preconditioned with 10 mL 1 M K_2_CO_3_ solution followed by 60 mL water for injection (Otsuka Pharmaceutical Factory, Naruto, Japan) and a Sep-Pak tC18 Plus Short Cartridge (Waters) preconditioned with 5 mL EtOH followed by 40 mL water for injection (Otsuka Pharmaceutical Factory) were installed between the VP34–VP35 and the VH17–VH18 positions, respectively.

**Table 1:** Details of module set-up for automated [^18^F]MC225 production with a CFN-MPS200 multipurpose synthesizer

Vial/component	Reagent
CFN-MPS200	
^18^O-water V-vial	Empty
^18^O-water recovery vial	Empty
R1	40 mM K_2_CO_3_ 0.5 mL + 15 mg K.222 in dry 2 mL MeCN
R2	1 mL dry MeCN
R3	20 mg 2-Bromoethyl tosylate in 1 mL *o*-DCB
R4	1mL water + 1 mL HPLC eluent
RV1	Empty
RV2	2 mg MC226 + 3 mg NaH + 0.5 mL DMF
QMA	Conditioned QMA
HPLC separation unit	
HPLC solvent	MeCN/50 mM AcONH_4_ sol. = 80/20 (500 mL)
HPLC column	Agilent Eclipse XDB-C18
Reservoir	Empty
Liq. Waste 1	Empty
Liq. Waste 2	Empty
SPE formulation unit	
sR1	10 mL water for injection
sR2	1.4 mL EtOH
sR3	1 mL ascorbate injection + 100 mL water for injection
Liq. Waste	Empty
Formulation vial	15 mL ascorbate injection/polysorbate 80/Sterile saline = 0.5/0.1/20
Product vial	Empty
tC18	Conditioned tC18
Sterile Filter	0.22 μm Millex GV

### Process description

**Fig. 2:**
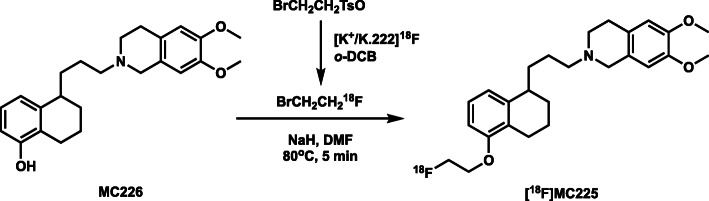
Radiosynthesis of [^18^F]MC225

[^18^F]MC225 was synthesized as previously reported by Savolainen et al. [29] via a two-pot reaction (Fig. 2). No-carrier-added ^18^F-fluoride was produced by bombardment of [^18^O]H_2_O (Taiyo Nippon Sanso, Tokyo, Japan) at 50 μA × 20 min with 20 MeV protons on a HM-20 cyclotron system (Sumitomo Heavy Industries). The target water containing ^18^F-fluoride was transferred directly to the V-vial. The irradiated [^18^O]H_2_O was passed through the QMA-cartridge connected between VP34 and VP35, and the enriched water was recovered in an ^18^O-water recovery vial. The QMA-trapped ^18^F-fluoride was eluted with a reagent of R1 [mixture of aqueous 0.5 mL of K_2_CO_3_ (20 μmol) and a 2.0 mL MeCN solution of K.222 (40 μmol)] into the RV1. The solvents were removed azeotropically with MeCN at 100 °C under a slight flow of N_2_ and vacuum over 15 min. This procedure was conducted once more with 1.0 mL MeCN from R2. The RV1 was then cooled to 40 °C prior to the next step. The reagent of R3 [20 mg of 2-bromoethyl tosylate (61 μmol) in 1.0 mL of *o*-DCB] was transferred to the RV1, and distillation of the formed 2-bromoethyl [^18^F]fluoride was started immediately at 90 °C with N_2_ gas flow to RV2 containing 2 mg of MC226 (5.2 μmol) and 3 mg of NaH (125 μmol) in 0.5 mL of DMF at room temperature. After radioactivity of RV2 reached a plateau (~ 7 min), RV2 was reacted for 5 min at 80 °C. After cooling to 40 °C, 2.0 mL of quenching solution in R4 was added and the reaction mixture was transferred to the reservoir of the HPLC separation unit. The product was separated by HPLC [column: Agilent Eclipse XDB-C18 (5 μm, 250 mm × 9.4 mm inner diameter; solvent: MeCN/50 mM AcONH_4_ = 65/35], at a flow rate of 5 mL/min. The eluent was monitored by UV 254 nm, and radioactivity detectors were connected in series. The fraction of [^18^F]MC225 (retention time = 9 min) was collected into dilution bottle sR3, which was preloaded with 100 mL water for injection (Otsuka Pharmaceutical Factory) containing 1 mL of 250 mg/mL ascorbate injection (Nipro Pharma, Osaka Japan). After mixing with N_2_, the solution was transferred to a tC18 cartridge connected between the VH17–VH18 positions. [^18^F]MC225 was trapped in the cartridge, which was washed with 10 mL of water for injection (sR1), and the product was eluted with 1.4 mL of EtOH (sR2) into a Formulation Vial preloaded with 15 mL of formulation buffer solution [ascorbate injection/polysorbate 80 (Fujifilm Wako Pure Chemical, Osaka Japan)/sterile saline (Otsuka Pharmaceutical Factory) = 0.5/0.1/20]. After gentle mixing with N_2_, the solution was transferred and passed through a 0.22-μm sterilizing filter (Millex GV; Merck Millipore, Darmstadt, Germany) into an empty 30 mL sterile vial (Mita Rika Kogyo, Osaka, Japan) fitted with a sterile-filtered venting needle (Terumo, Tokyo, Japan).

### Quality control

Filter integrity was assessed by bubble point test (SLTEST000; Merck Millipore). The pH value of the injection solution was determined using a pH meter (LAQUA F-72; Horiba, Kyoto, Japan). Residual solvents in the injection solution were measured by capillary gas chromatography using a GC-2014 system with LabSolutions Software (Shimadzu, Kyoto, Japan). The radionuclide was identified by determining the half-life measurement using a Capintec CRC-55tR dose calibrator (Florham Park, NJ, USA) and the measurement of the 511 keV photopeak using a NaI(Tl) radiation detector (US-2000; Universal Giken, Odawara, Japan). Residual K.222 in the injection solution was evaluated by spot test. The limulus amebocyte lysate (LAL) test was conducted on a Toxinometer® ET-6000 (Fujifilm Wako Pure Chemical). Finally, a sample of the product formulation was tested for sterility post-release using direct inoculation in accordance with the Japanese Pharmacopoeia, 17th edition.

HPLC analysis was performed on a Shimadzu Prominence HPLC system equipped with a model LC-20AD pump, model SPD-20A UV absorbance detector (set at 280 nm), a GABI 3 × 3 in. NaI scintillation detector (Elysia-Raytest, Straubenhardt, Germany), and an analytical column (YMC-Pack ODS-A, 3 μm, 50 mm × 2.1 mm inner diameter) purchased from YMC (Kyoto, Japan). Operation of the Shimadzu Prominence HPLC system was controlled using Shimadzu LabSolutions software. For analysis, isocratic elution was applied using MeCN/50 mM AcONH_4_ solution = 60/40 (flow rate = 1 mL/min). Retention time of the authentic standard MC225 was 7.6 min.

### Acute toxicity

Toxicity studies of MC225 were performed at the Kannami Laboratory, BoZo Research Center (Shizuoka, Japan). Acute toxicity was assayed in Sprague-Dawley rats [Crl:CD(SD)]. MC225 at a dose of 2.5 mg/kg bodyweight (0.5 mg/mL in 10 w/v% DMSO containing water for injection) was injected intraperitoneally into 6-week-old rats weighing 226–236 g (males, *n* = 5) and 149–161 g (females, *n* = 5). The dose of 2.5 mg/kg bodyweight is the 10,000-fold equivalent of the postulated maximum administration dose (0.25 μg/kg bodyweight) of 370 MBq [^18^F]MC225, with the lowest molar activity of 6.3 MBq/nmol for humans weighing 40 kg. Rats were observed frequently until 30 min and then at 1, 2, 4, and 6 h after the injection on day 1, and thereafter once daily for 14 days for clinical signs of toxicity. Rats were weighed on days 1, 2, 4, 7, and 14. At the end of the 14-day observation period, the rats were euthanized by exsanguination under isoflurane anesthesia, and a macroscopic analysis of the autopsy samples was performed. Three batches of [^18^F]MC225 were prepared and assayed after the decay-out of ^18^F. Solutions with decayed [^18^F]MC225 were individually injected intravenously into 6-week-old male and female rats (*n* = 3 each) at doses of 13.15 μg/5.29 mL/kg bodyweight, 9.22 μg/4.63 mL/kg bodyweight, and 1.68 μg/3.91 mL/kg bodyweight, for each of the three batches, equivalent to 200-fold of the postulated administration dose of 185 MBq [^18^F]MC225 for humans. After injection of decayed [^18^F]MC225, the rats were observed for clinical signs of toxicity for 14 days, and a macroscopic analysis was then performed as described above.

### Mutagenicity

Mutagenicity tests were performed at the Tokyo Laboratory, BoZo Research Center (Tokyo, Japan). MC225 was tested for mutagenicity by the Ames test with four histidine-requiring strains of *Salmonella typhimurium* (TA98, TA100, TA1535, and TA1537) and one strain of *Escherichia coli* (WP2*uvr*A), with and without the S9 mixture, at a dose range of 19.5–5000 μg/plate according to the standard method.

### Dosimetry

[^18^F]MC225 (2.1 MBq/1.6 pmol) was injected intravenously into 8-week-old male ddY mice. The tracer-injected mice were housed individually in filter-paper-lined animal-rearing cages until the time of euthanasia. Mice were killed by cervical dislocation at 5, 15, 30, 60, 180, and 360 min after injection (*n* = 4 each). The blood was collected by heart puncture, and the tissues were harvested. Radioactivity excreted into the urine was recovered from the cage floor and by cystocentesis from the urinary bladder. The samples were measured for ^18^F radioactivity with an auto-gamma counter (Hidex-AMG, Turk, Finland) and weighed. The tissue uptake of ^18^F was expressed as the percentage of injected dose per organ (%ID/organ) or the percentage of injected dose per gram of tissue (%ID/g). The tissue distribution data were extrapolated to an adult male phantom using the %kg/g method [34]. The radiation absorbed dose and effective dose for human adults were estimated using OLINDA/EXM software (Vanderbilt University, Nashville, TN, USA) [35].

## Results

### Automated synthesis

**Fig. 3:**
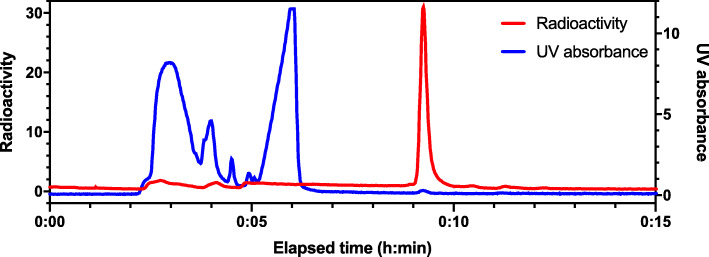
Representative preparative HPLC chromatogram of the reaction mixture

The three production runs had activity yields of 3527 ± 965 MBq, decay-corrected yields of 12.8 ± 2.6%, molar activity of 1576 ± 446 GBq/μmol, and radiochemical purity of 99.5 ± 0.3% (Table 2). The average synthesis time following target bombardment was 74 min. All batches of [^18^F]MC225 injection met the QC criteria listed in Table 2. [^18^F]MC225 was stable for up to 2 h after end of synthesis, with acceptable appearance, pH of 6.2 ± 0.1, and radiochemical purity of 98.3 ± 0.3%. Figure 3 shows a representative semi-preparative HPLC chromatogram of the reaction mixture. The phenol precursor (MC226), which elutes at approximately 6 min, was well separated from the product (MC225), which elutes at approximately 9 min.

**Table 2:** Product release specifications and validation test results of [^18^F]MC225

QC test	Release criteria*	Run 1	Run 2	Run 3
Visual inspection	Clear, colorless to slightly yellow solution, free of particulate matter	Pass	Pass	Pass
Radiochemical identity	Retention time of [^18^F]MC225 peak within ± 10% min compared with the retention time of the known reference MC225 peak	7.1%	4.4%	4.3%
Radionuclide identity by 511 keV peak	Peak energy of gamma ray spectrum at 511 keV	Pass	Pass	Pass
Radionuclide identity by half-life determination	105–115 min	110 min	108 min	115 min
Content of ethanol	≤ 10% (v/v)	7.3%	6.7%	6.6%
Residual acetonitrile	≤ 410 μg/mL	36.8 μg/mL	75.4 μg/mL	121.7 μg/mL
Residual dimethylformamide	≤ 880 μg/mL	N.D.	N.D.	N.D.
Residual o-dichlorobenzene	≤ 25 μg/mL	9.35 μg/mL	5.41 μg/mL	3.86 μg/mL
Content of MC225	≤ 10μg/dose	0.5 μg/dose	0.6 μg/dose	0.9 μg/dose
Total content of MC225 and related impurities	≤ 10μg/dose	5.4 μg/dose	6.2 μg/dose	3.3 μg/dose
pH	6.0 to 8.5	6.2	6.2	6.3
Radiochemical purity at release	≥ 90%	98.7%	99.2%	99.6%
Molar activity at end of synthesis	N/A	1613 GBq/μmol	2002 GBq/μmol	1112 GBq/μmol
Residual K.222	≤ 40 μg/mL	Pass	Pass	Pass
Bacterial endotoxins	< 150 EU/vial	< 48.5 EU	< 53.3 EU	< 51.3 EU
Sterility testing	No growth observed in 14 days	Pass	Pass	Pass
Filter integrity by bubble point testing	≥ 40 psi	48 psi	44 psi	44 psi

*Release criteria apply to a maximum administration volume of 10 mL

### Acute toxicity

Acute toxicity in rats was evaluated after a single intraperitoneal injection of MC225 at a dose of 2.5 mg/kg and a single intravenous injection of one of the three lots of [^18^F]MC225 preparations at a dose range of 1.68–13.15 μg/kg. There was no mortality in the rats during the 14-day observation period. All rat groups showed normal gains in bodyweight compared with the control animals, and no clinical signs of toxicity were observed over the 15-day period. Postmortem macroscopic examination found no abnormalities.

### Mutagenicity

A bacterial reverse mutation test conducted using *Salmonella thyphimurium* and *Escherichia coli* detected no mutagenic activity for MC225.

### Dosimetry

**Fig. 4:**
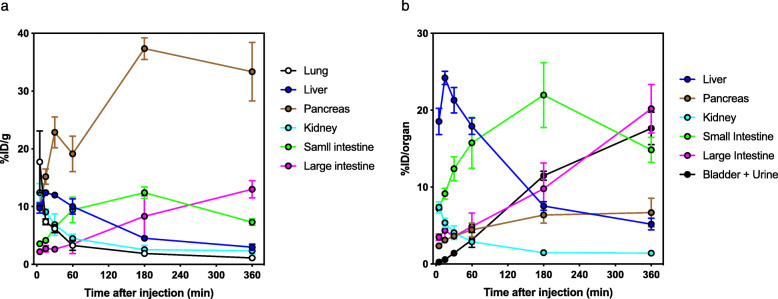
Regional decay-corrected time activity curves showing high radioactivity concentration (**a**) and accumulation (**b**) after intravenous injection of [^18^F]MC225 into mice. Data are presented as the mean ± S.D. (*n* = 4)

The tissue distribution of radioactivity after injection of [^18^F]MC225 into mice is summarized in Fig. 4, and in Tables 3 and 4. The radioactivity concentrations in the blood decreased rapidly after [^18^F]MC225 injection. The lung and kidney showed initial high uptake (%ID/g) before decreasing gradually (Fig. 4a). Among all of the examined organs, the pancreas showed the highest radioactivity concentration, reaching 37%ID/g at 180 min, which was maintained at 33%ID/g for 360 min after injection (Fig. 4a). Excretion of radioactivity into the bladder and urine increased gradually in response to the clearance of radioactivity from the kidney, reaching 18%ID/organ (bladder + urine) at 360 min after injection (Fig. 4b). Radioactivity of the liver peaked (24%ID/organ) at 15 min after injection, before clearing (Fig. 4b). In response to the clearance of radioactivity from the liver, radioactivity levels of the small intestine peaked at 180 min (22%ID/organ), and radioactivity of the large intestine gradually increased to reach 20%ID/organ at 360 min after injection (Fig. 4b). These data demonstrate that radioactivity was excreted by both the hepatobiliary and renal urinary systems. The radiation absorbed dose was estimated from these biodistribution data (Table 5). The absorbed dose (μGy/MBq) calculated with urination at 360 min after injection was highest in the pancreas (63.8), small intestine (36.0), urinary bladder wall (32.5), and upper large intestine wall (30.4). The absorbed dose calculated without urination was highest in the pancreas (63.9), urinary bladder wall (59.6), small intestine (36.3), and upper large intestine wall (30.6). The effective dose according to the risk-weighting factors of ICRP103 [36] was estimated as 15.7 μSv/MBq (with urination at 360 min after injection) and 16.9 μSv/MBq (without urination).

**Table 3:** Tissue distribution of radioactivity in mice after intravenous injection of [^18^F]MC225

	Injected dose/g tissue (%) *					
	5 min	15 min	30 min	60 min	180 min	360 min
Blood	0.70 ± 0.09	0.55 ± 0.19	0.73 ± 0.13	0.87 ± 0.25	0.97 ± 0.09	0.64 ± 0.04
Heart	2.82 ± 0.32	1.54 ± 0.10	1.54 ± 0.23	1.23 ± 0.29	1.28 ± 0.12	0.86 ± 0.12
Lung	17.73 ± 5.34	7.35 ± 0.49	6.17 ± 0.71	3.28 ± 0.87	1.88 ± 0.12	1.11 ± 0.13
Liver	9.74 ± 0.86	12.41 ± 0.28	11.99 ± 0.19	10.01 ± 1.31	4.51 ± 0.21	2.98 ± 0.51
Pancreas	10.12 ± 0.29	15.17 ± 1.32	22.85 ± 2.69	19.13 ± 3.08	37.36 ± 1.87	33.34 ± 5.05
Spleen	4.85 ± 0.77	4.34 ± 1.04	4.83 ± 0.30	2.64 ± 0.62	2.24 ± 0.39	0.94 ± 0.17
Kidney	12.40 ± 1.55	9.06 ± 0.50	6.88 ± 1.88	4.46 ± 0.73	2.55 ± 0.78	2.35 ± 0.56
Stomach	3.14 ± 0.64	3.03 ± 0.18	3.66 ± 1.02	5.69 ± 1.10	3.90 ± 1.35	2.69 ± 0.75
Small intestine	3.57 ± 0.24	4.13 ± 0.24	6.37 ± 0.75	9.43 ± 2.22	12.40 ± 1.01	7.30 ± 0.55
Large intestine	2.19 ± 0.32	2.70 ± 0.55	2.62 ± 0.15	3.52 ± 1.68	8.33 ± 3.57	13.00 ± 1.50
Testis	0.91 ± 0.28	0.87 ± 0.11	1.16 ± 0.08	1.11 ± 0.31	1.76 ± 0.14	1.49 ± 0.27
Muscle	1.84 ± 0.30	1.26 ± 0.17	1.33 ± 0.05	1.28 ± 0.43	0.80 ± 0.05	0.50 ± 0.09
Bone	1.65 ± 0.12	1.51 ± 0.16	2.16 ± 0.15	1.52 ± 0.22	2.46 ± 0.17	3.38 ± 1.03
Brain	1.24 ± 0.09	1.27 ± 0.29	1.14 ± 0.04	0.93 ± 0.11	0.81 ± 0.05	0.53 ± 0.05

*Mean ± S.D. (*n* = 4)

**Table 4:** Organ distribution of radioactivity in mice after intravenous injection of [^18^F]MC225

	Injected dose/organ (%) *					
	5 min	15 min	30 min	60 min	180 min	360 min
Heart	0.44 ± 0.06	0.26 ± 0.00	0.22 ± 0.01	0.22 ± 0.03	0.18 ± 0.01	0.14 ± 0.01
Lung	3.48 ± 0.93	1.61 ± 0.05	1.08 ± 0.12	0.79 ± 0.06	0.35 ± 0.03	0.21 ± 0.06
Liver	18.55 ± 1.70	24.21 ± 0.86	21.31 ± 1.66	17.92 ± 1.07	7.53 ± 0.57	5.18 ± 0.75
Pancreas	2.35 ± 0.27	3.10 ± 0.33	3.62 ± 0.28	4.44 ± 0.88	6.37 ± 1.05	6.68 ± 1.86
Spleen	0.55 ± 0.12	0.48 ± 0.07	0.47 ± 0.03	0.34 ± 0.08	0.17 ± 0.04	0.08 ± 0.01
Kidney	7.36 ± 0.70	5.36 ± 0.59	4.07 ± 0.89	2.88 ± 0.34	1.47 ± 0.32	1.40 ± 0.35
Stomach	2.57 ± 0.41	3.36 ± 0.38	3.02 ± 0.52	3.80 ± 0.61	2.60 ± 0.60	2.01 ± 0.51
Small intestine	7.20 ± 0.52	9.15 ± 0.71	12.38 ± 1.57	15.76 ± 3.32	21.98 ± 4.21	14.85 ± 1.62
Large intestine	3.50 ± 0.41	4.35 ± 1.10	3.58 ± 0.29	4.87 ± 1.75	9.79 ± 3.34	20.19 ± 2.26
Testis	0.20 ± 0.03	0.29 ± 0.04	0.28 ± 0.03	0.33 ± 0.07	0.42 ± 0.04	0.39 ± 0.09
Brain	0.50 ± 0.05	0.54 ± 0.14	0.45 ± 0.01	0.40 ± 0.06	0.31 ± 0.03	0.23 ± 0.02
Bladder	0.13 ± 0.08	0.12 ± 0.03	0.06 ± 0.02	0.26 ± 0.15	0.12 ± 0.10	0.38 ± 0.20
Urine	0.10 ± 0.01	0.43 ± 0.19	1.34 ± 0.25	2.88 ± 0.88	11.35 ± 0.57	17.26 ± 2.26
Bladder + urine	0.24 ± 0.07	0.56 ± 0.16	1.40 ± 0.26	3.13 ± 0.97	11.48 ± 0.60	17.64 ± 2.11

*Mean ± S.D. (*n* = 4)

**Table 5:** Absorbed dose of [^18^F]MC225 for human adults estimated from mouse data

Target organ	Absorbed dose (μGy/MBq)	
	With urination*	Without urination
Adrenals	12.9	12.9
Brain	5.0	5.0
Breasts	7.8	7.8
Gallbladder	16.0	16.1
Lower large intestine wall	28.9	29.7
Small intestine	36.0	36.3
Stomach wall	19.5	19.5
Upper large intestine wall	30.4	30.6
Heart wall	10.3	10.3
Kidneys	18.0	18.1
Liver	26.9	26.9
Lungs	13.4	13.4
Muscle	10.2	10.4
Ovaries	15.5	16.2
Pancreas	63.8	63.9
Red marrow	12.0	12.2
Osteogenic cells	14.8	14.9
Skin	7.1	7.1
Spleen	13.9	13.9
Testes	8.5	9.0
Thymus	9.3	9.3
Thyroid	9.2	9.2
Urinary bladder wall	32.5	59.6
Uterus	15.3	17.0
Total body	11.9	12.1
Effective dose (μSv/MBq)	15.7	16.9

*Urination at 360 min after injection

## Discussion

We automated the radiolabeling process of [^18^F]MC225 on a CFN-MPS200 multipurpose synthesizer. The obtained activity yields (2650–4561 MBq) and high molar activity (1112–2002 GBq/μmol) were good enough for clinical research purposes. We started the radiosynthesis from approximately 40 GBq of ^18^F-fluoride (QMA-trapped radioactivity). It is possible to further improve activity yields and molar activity by starting with higher levels of ^18^F-fluoride radioactivity. The high molar activity of [^18^F]MC225 probably resulted from using fluoride-free materials (e.g., silicon tubes, thermoplastic elastomer tubes, polypropylene connectors) in assembly of the cassette of the CFN-MPS200. All three validation runs of [^18^F]MC225 satisfied the QC release criteria. No degradation of [^18^F]MC225 in formulation was confirmed up to 2 h. Taken together, the radiolabeling process of [^18^F]MC225 on a CFN-MPS200 was considered to be of sufficient quality for clinical use. This synthesis process has been approved for human use by the Institutional PET Drug Committee and MHLW Certified Clinical Research Review Board, Tokyo Metropolitan Geriatric Medical Center (CRB3180026) and is now in use for first-in-human clinical research (jRCTs031190136).

The absence of any abnormality in rats in the acute toxicity test together with the absence of mutagenicity of MC225 demonstrated the clinical suitability of [^18^F]MC225 for PET studies in humans. The estimated NOAEL of MC225 is > 5.85 μmol/2.5 mg/kg, which is the 10,000–fold equivalent of the postulated maximum administration dose. Because PET tracers are administered intravenously as a bolus, it is important to evaluate their potential toxic hazard to the cardiovascular system. Fusi et al. evaluated the effect of MC225 on the mechanical activity of freshly isolated rat aortic rings and on Ca_v_1.2 channel current (*I*_Ca1.2_) of A7r5 cells, as well as on cardiac function and electrocardiogram in Langendorff-perfused isolated rat hearts [37]. These in vitro cardiovascular studies demonstrated that MC225 has potential to induce vasodilator action and cardiotoxic effect over the 1000-fold equivalent postulated maximum administration dose (0.58 nmol/0.25 μg/kg) of MC225. All three validation runs demonstrated high molar activity of [^18^F]MC225 (> 1000 GBq/μmol) and a low amount of MC225-related chemical impurities. Because the estimated total chemical contents are ≤ 1.5 μg/185 MBq, the potential risk associated with [^18^F]MC225 injection is considered to be within the toxicologically acceptable range [38].

The radiation absorbed dose was highest in the pancreas, followed by the urinary bladder, small intestine wall, and large intestine wall. Urination at 360 min after injection significantly decreased the absorbed dose in the urinary bladder. Except for the pancreas, all of the organs with high absorbed dose are in the excretion route. The effective dose was well within the previously reported range (15–30 μSv/MBq) for ^18^F-labeled PET radiopharmaceuticals [39]. In the case of administration of 185 MBq of [^18^F]MC225, effective dose is estimated as 2.9 mSv (with urination at 360 min after injection) and 3.1 mSv (without urination), which is within the strict limit of 10 mSv set by the ICRP recommendations [40] and practiced in Europe. In this condition, the highest absorbed dose in the pancreas (with or without urination) is estimated as 11.8 mGy, which is also within the strict limits for individual organs (30 mSv for sensitive organs and 50 mSv for all others), as required by US Radioactive Drug Research Committee regulations [41].

There have been several reports indicating that preclinical (i.e., animal-derived) dosimetry of ^18^F-labeled tracers underestimates 20–40% of the effective dose to humans [42]. Taking into account the discrepancy between extrapolated and actually determined data in humans, we calculated effective dose of [^18^F]MC225 dividing by 0.6 to correct 40% underestimation to humans. The corrected dose (26.1 and 28.1 μSv/MBq for urination and without urination, respectively) was still within the previously reported ranges of ^18^F-labeled PET radiopharmaceuticals [39]. Furthermore, administration of 185 MBq of [^18^F]MC225 was still within the strict limit of 10 mSv (4.8 and 5.2 mSv for urination and without urination, respectively).

## Conclusion

The automated synthesis of [^18^F]MC225 for clinical use was successfully and efficiently achieved on a CFN-MPS200 multipurpose synthesizer. All three process validation batches complied with the product specifications, and the process was confirmed to be appropriate for the production of [^18^F]MC225. Preclinical toxicological studies indicated that [^18^F]MC225 shows acceptable pharmacological safety at the dose required for adequate PET imaging. Taking into account the high molar activity and low total chemical contents, [^18^F]MC225 will be tolerable in first-in-human clinical trials. A single intravenous injection of 185 MBq of [^18^F]MC225 leads to an estimated effective dose of 2.9 mSv (with urination at 360 min after injection) and 3.1 mSv (without urination), and highest absorbed dose to an organ of 11.8 mGy (with or without urination). The potential risk associated with [^18^F]MC225 PET imaging is well within acceptable dose limits.

## Data Availability

The datasets used and/or analyzed during the current study are available from the corresponding author on reasonable request.

## Abbreviations

AcONH_4_: Ammonium acetate
BBB: Blood-brain barrier
CNS: Central nervous system
*o*-DCB: *o*-Dichlorobenzene
DMF: *N*, *N*-Dimethylformamide
DMSO: Dimethyl sulfoxide
EtOH: Ethanol
EU: Endotoxin unit
HPLC: High performance liquid chromatography
ICRP: International Commission on Radiological Protection
K.222: 4,7,13,16,21,24-Hexaoxa-1,10-diazabicyclo[8.8.8]hexacosane
LAL: Limulus amebocyte lysate
MeCN: Acetonitrile
MC225: 5-(1-(2-fluoroethoxy))-[3-(6,7-dimethoxy-3,4-dihydro-1H-isoquinolin-2-yl)-propyl]-5,6,7,8-tetrahydronaphthalen
MC226: 5-(1-(2-hydroxy))-[3-(6,7-dimethoxy-3,4-dihydro-1H-isoquinolin-2-yl)-propyl]-5,6,7,8-tetrahydronaphthalen
MHLW: Ministry of Health, Labor and Welfare
N/A: Not applicable
N.D: Not detected
NOAEL: No observable adverse effect level
%ID/g: Percentage of injected dose per gram of tissue
%ID/organ: Percentage of injected dose per organ
PET: Positron emission tomography
P-gp: P-glycoprotein
QC: Quality control
Verapamil: 2-(3,4-dimethoxyphenyl)-5-[2-(3,4-dimethoxyphenyl)ethyl-methylamino]-2-propan-2-ylpentanenitrile
SPE: Solid phase extraction
S.D.: standard deviation
UV: Ultraviolet
